# Comprehensive immunohistochemical analysis of PD-L1 shows scarce expression in castration-resistant prostate cancer

**DOI:** 10.18632/oncotarget.22888

**Published:** 2017-12-04

**Authors:** Christian D. Fankhauser, Peter J. Schüffler, Silke Gillessen, Aurelius Omlin, Niels J. Rupp, Jan H. Rueschoff, Thomas Hermanns, Cedric Poyet, Tullio Sulser, Holger Moch, Peter J. Wild

**Affiliations:** ^1^ Department of Urology, University Hospital Zurich, University of Zurich, Zurich, Switzerland; ^2^ Department of Computer Science, ETH Zurich, Zurich, Switzerland; ^3^ Department of Medical Physics, Memorial Sloan Kettering Cancer Center, New York, NY, USA; ^4^ Department of Medical Oncology and Hematology, Cantonal Hospital, St. Gallen, Switzerland; ^5^ Department of Pathology and Molecular Pathology, University Hospital Zurich, University of Zurich, Zurich, Switzerland

**Keywords:** prostate cancer, PD-L1, immunotherapy, immune response

## Abstract

**Background:**

We aimed to analyze the frequency and distribution of PD-L1 expression in specimens from prostate cancer (PC) patients using two different anti-PD-L1 antibodies (E1L3N, SP263).

**Materials and Methods:**

PD-L1 immunohistochemistry was performed in a tissue microarray consisting of 82 castration-resistant prostate cancer (CRPC) specimens, 70 benign prostate hyperplasia (BPH) specimens, 96 localized PC cases, and 3 PC cell lines, using two different antibodies (clones E1L3N, and SP263). Staining images for CD4, CD8, PD-L1, and PanCK of a single PD-L1 positive case were compared, using a newly developed dot-wise correlation method for digital images to objectively test for co-expression.

**Results:**

Depending on the antibody used, in tumor cells (TC) only five (E1L3N: 6%) and three (SP263: 3.7%) samples were positive. In infiltrating immune cells (IC) 12 (SP263: 14.6%) and 8 (E1L3N: 9.9%) specimens showed PD-L1 expression. Two PC cell lines (PC3, LnCaP) also displayed membranous immunoreactivity. All localized PCs or BPH samples tested were negative. Dot-wise digital correlation of expression patterns revealed a moderate positive correlation between PD-L1 and PanCK expression, whereas both PanCK and PD-L1 showed a weak negative Pearson correlation coefficient between CD4 and CD8.

**Conclusions:**

PD-L1 was not expressed in localized PC or BPH, and was only found in a minority of CRPC tumors and infiltrating immune cells. Protein expression maps and systematic dot-wise comparison could be a useful objective way to describe the relationship between immuno- and tumor-related proteins in the future, without the need to develop multiplex staining methods.

## INTRODUCTION

The therapeutic options for castration-resistant prostate cancer (CRPC) have increased dramatically in the past years. Recent clinical trials have led to the FDA approval of cabazitaxel [1], abiraterone [2–4], enzalutamide [5, 6], radium-223 [7], and Sipuleucel-T [8]. Nevertheless, most CRPC patients develop resistance to these new agents, making it of utmost importance to develop newer treatment strategies [9]. The objective responses to immunotherapy in other cancers are driving renewed enthusiasm for cancer immunotherapy, however, the role of immunotherapy in CRPC is yet to be fully elucidated [10].

Programmed Death Receptor 1 (PD-1, or CD279), a member of the extended family of T cell regulators, is expressed on the surface of activated T cells, B cells, and macrophages [12]. Its ligand, Programmed Death Receptor Ligand 1 (PD-L1, or B7-H1 or CD274), is expressed on tumor cells, macrophages, T cells and certain other cell types [12], and the interaction of these two molecules negatively regulates immune responses. Of major interest is that inhibition of the interaction between PD1 and PD-L1 can enhance T-cell responses *in vitro,* and mediates clinical anti-tumor activity [13–20]. A correlation between PD-L1 expression on tumor or immune cells in the tumor specimen and tumor response to anti-PD1 or anti-PD-L1 immunotherapy has been described in various advanced tumors [13–21]. PD-L1 is expressed in a wide range of tumors, at a frequency of up to 88% in some types of cancer [22]. In the tumor microenvironment, PD-L1 expressed on tumor cells binds to PD-1 on activated T cells that have migrated to the tumor. This delivers an inhibitory signal to those T cells, preventing them from killing target tumor cells, and protecting the tumor from immune elimination [22]. Recently, in a small study with ten CRPC patients treated with pembrolizumab, response in three patients has been reported [23]. Tumor tissue was available for two of these three patients, and showed PD-L1 expression. In addition, primary and metastatic CRPC showed robust synergistic responses when immune checkpoint blockade was combined with myeloid-derived suppressor cells (MDSC)-targeted therapy. Mechanistically, combination therapy efficacy stemmed from the upregulation of interleukin-1 receptor antagonist and suppression of MDSC-promoting cytokines secreted by prostate cancer cells. These recent observations by Lu et al. illuminate a new treatment concept, combining immune checkpoint blockade with MDSC-targeted therapies for mCRPC [11].

The first aim of this study was to systematically describe the expression of PD-L1 in benign prostatic hyperplasia (BPH), localized prostate cancer (PC), and CRPC using two anti-PD-L1 antibodies. The second aim was to observe if PD-L1 status was dependent on previous treatment modalities. The third aim was to develop a new method to describe the relationship between immune cells and tumor-related proteins using pseudo-colored protein expression maps, and to use dot-wise correlation coefficients of superimposed images as an objective co-location measurement.

## RESULTS

### Expression of PD-L1 in benign and malignant prostate tissue

**Figure 1:**
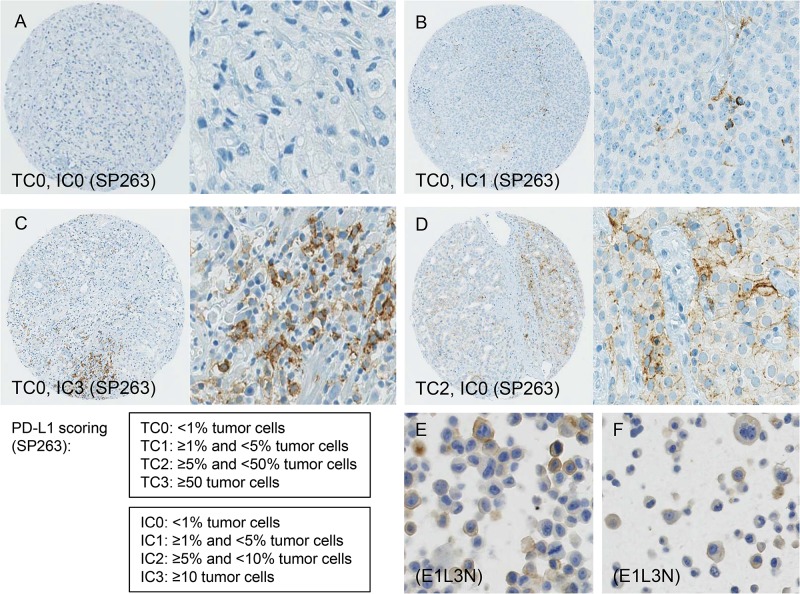
(**A–F**) Programmed death ligand 1 (PD-L1) scoring criteria and examples of tumor cell (TC) and immune cell (IC) staining in CRCP specimens and cell lines (clone SP263 and E1L3N). A: negative PD-L1 immunoreactivity in a CRCP specimen. B: PD-L1 staining in >1% of immune cells in a CRPC sample. C: PD-L1 expression in >30% of immune cells.. D: PD-L1 expression in > 5% of tumor cells. E, F: staining of PD-L1 in formalin-fixed paraffin-embedded PC3 (E) and LnCaP (F) cell culture pellets. Diameter of tissue microarray spot 0.6 mm. Inserts and cell line images, original magnification 400x. Scoring criteria of PD-L1 immunohistochemistry according to Spira et al. [43].

**Figure 2:**
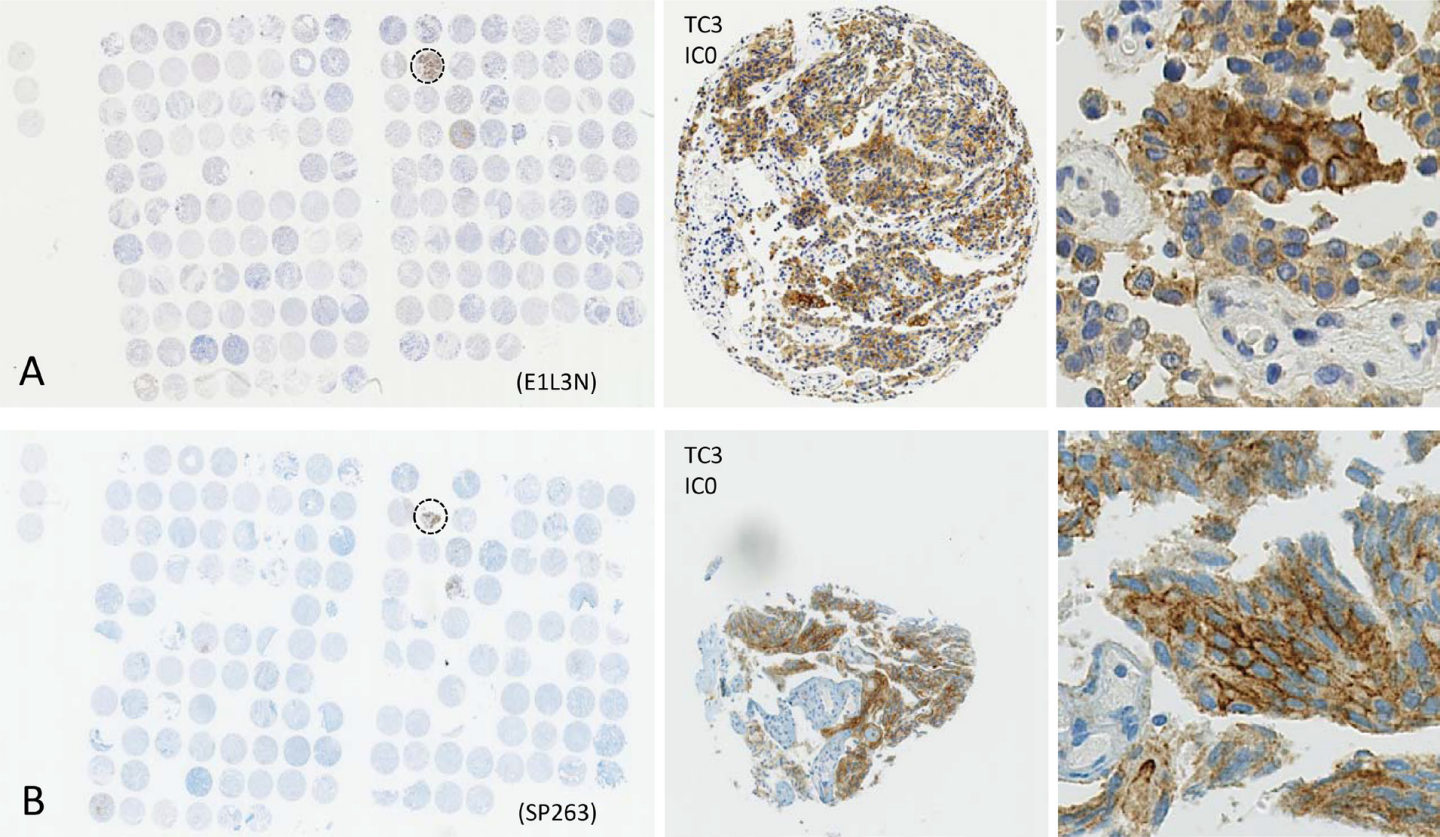
Comparison of PD-L1 staining results for two different monoclonal anti-PD-L1 antibodies using tissue microarrays of CRPC specimens (panel (**A**) E1L3N, panel (**B**) SP263). Single tissue spots have a diameter of 0.6 mm. Zoomed versions, showing positive membranous PD-L1 immunoreactivity in a positive CRCP patient (dashed circle).

**Figure 3:**
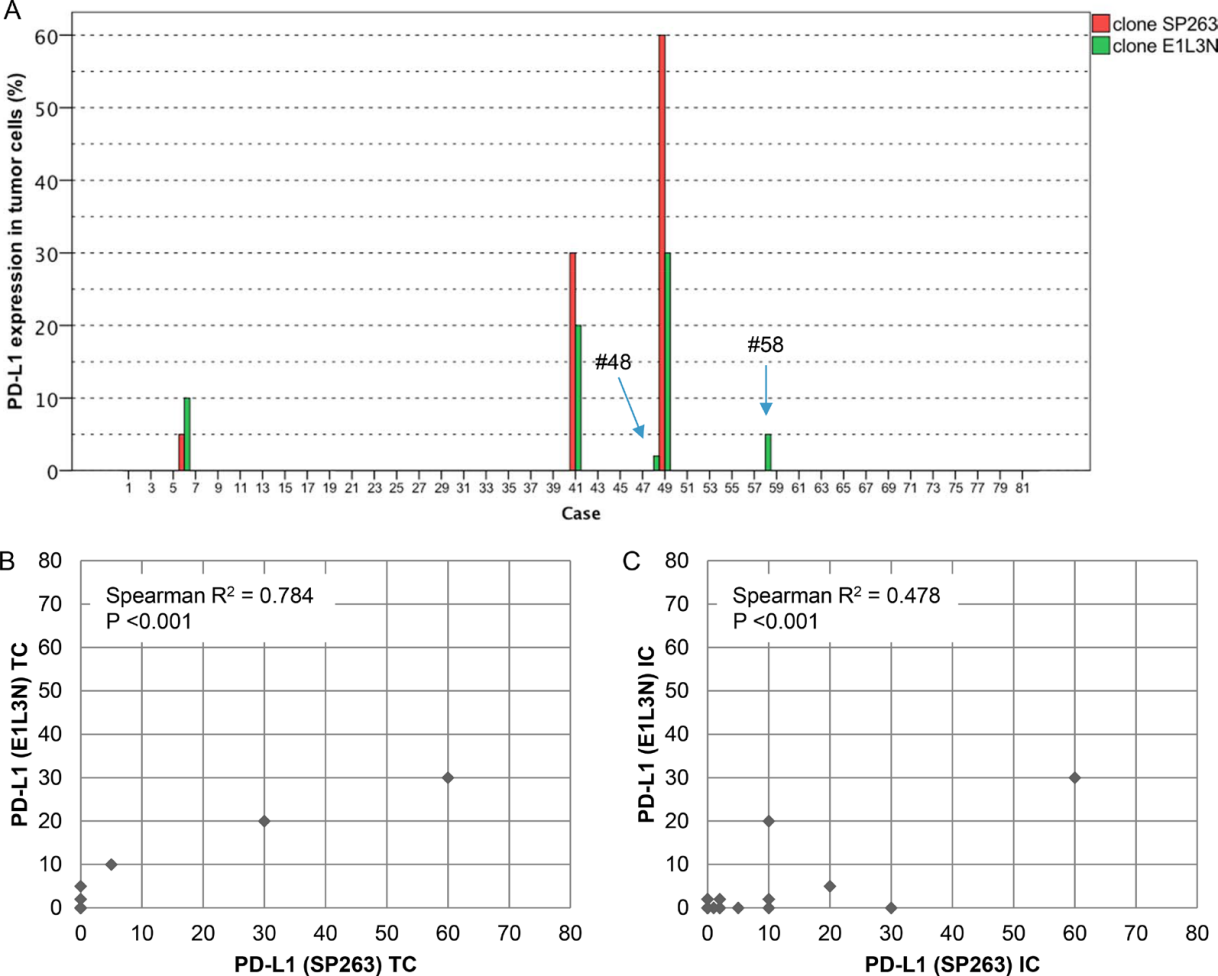
Direct comparison of anti-PD-L1 antibodies E1L3N and SP263 (**A**) Direct expression values (% positive tumor cells) for clone E1L3N (green) and SP263 (red). (**B** and **C**) Scatter plots for the comparison of immunoreactivity in % (clones E1L3N, SP263) regarding tumor cells (3B) and immune cells (3C).

**Figure 4:**
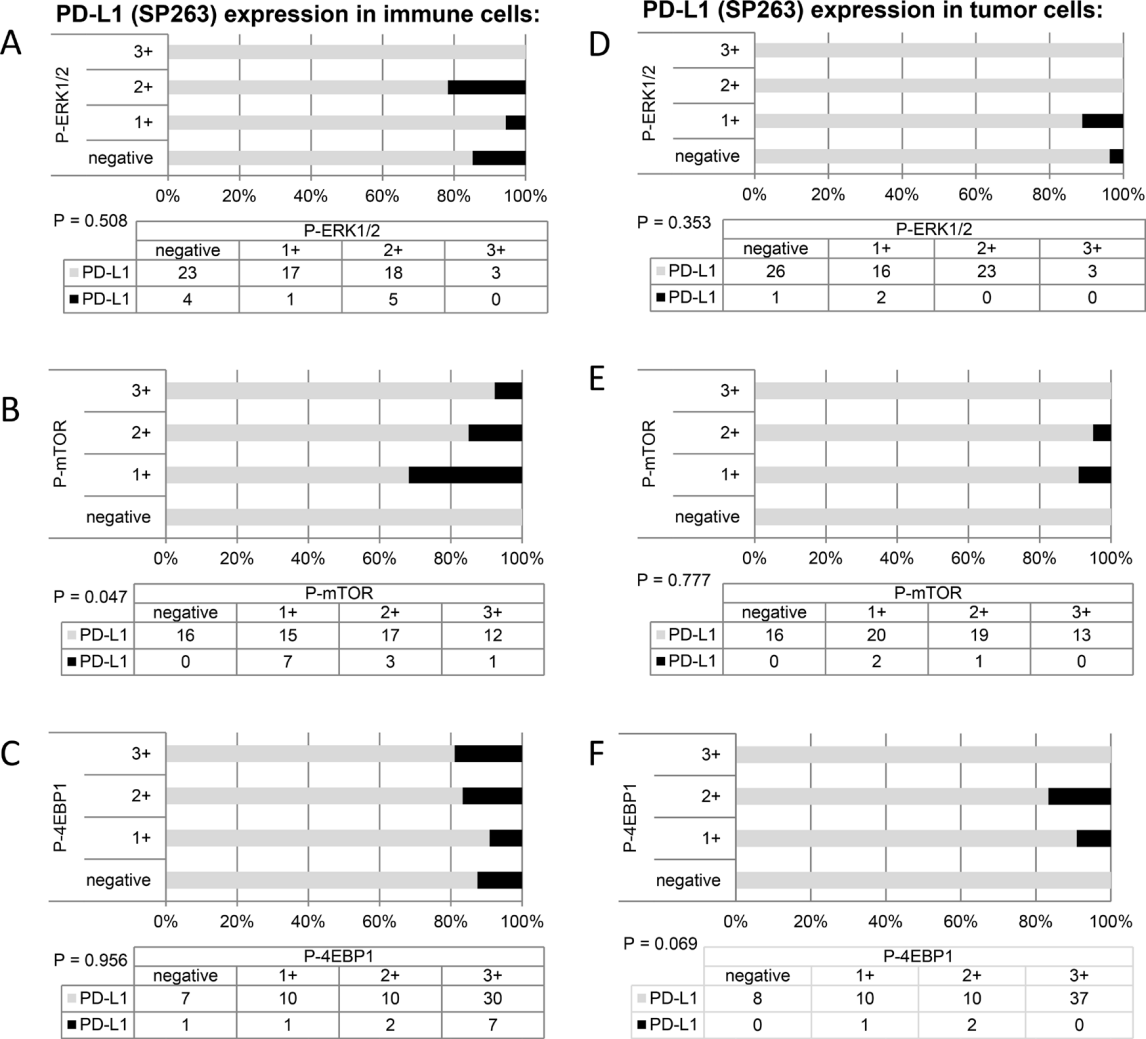
Cumulative bar charts showing the association of PD-L1 immunoreactivity with the expression of phospho-ERK1/2, phospho-mTOR, and phospho-4E-BP1

This study included 248 tissue samples (70 BPH, 96 PC, 82 CRPC) and 3 PC cell lines. There was no expression of PD-L1 observed in either BPH or localized PC samples (0%). Examples of PD-L1 tumor cell (TC) and immune cell (IC) staining in CRCP specimens and cell lines (clone SP263 and E1L3N) are given in Figure 1A–1F. Of the three tested paraffin embedded cell cultures (E1L3N), only PC3 and LnCaP showed membranous PD-L1 expression. A comparison of staining patterns and scoring was performed on serial sections of tissue microarrays with CRPC samples (Figure 2A–2B). Two antibody clones were used for PD-L1 immunohistochemistry assays (E1L3N, SP263). Overall, PD-L1 expression patterns were similar in both assays, and CRPC samples displayed heterogeneous PD-L1 expression. As illustrated in Figure 2B, clone SP263 showed the strongest membranous staining in tumor cells. However, with clone SP263, only three of 82 analyzable cases showed membranous immunoreactivity in more than 1% of tumor cells (3.7%), whereas clone E1L3N revealed 5 positive out of 81 analyzable CRCP samples (6.0%). Figure 3A displays the direct expression values (% positive tumor cells) by using clone E1L3N *versus* SP263. The comparison of PD-L1 expression (%) with clone SP263 versus E1L3N showed significant correlation coefficients (Figure 3B and 3C). No significant association of PD-L1 immunoreactivity with expression of phospho-ERK1/2 (Figure 4A and 4D), phospho-mTOR (Figure 4B and 4E), phospho-4E-BP1 (Figure 4C and 4F), and Ki-67 proliferation fraction (data not shown) could be observed, after correction for multiple testing (Figure 4A–4F).

**Figure 5:**
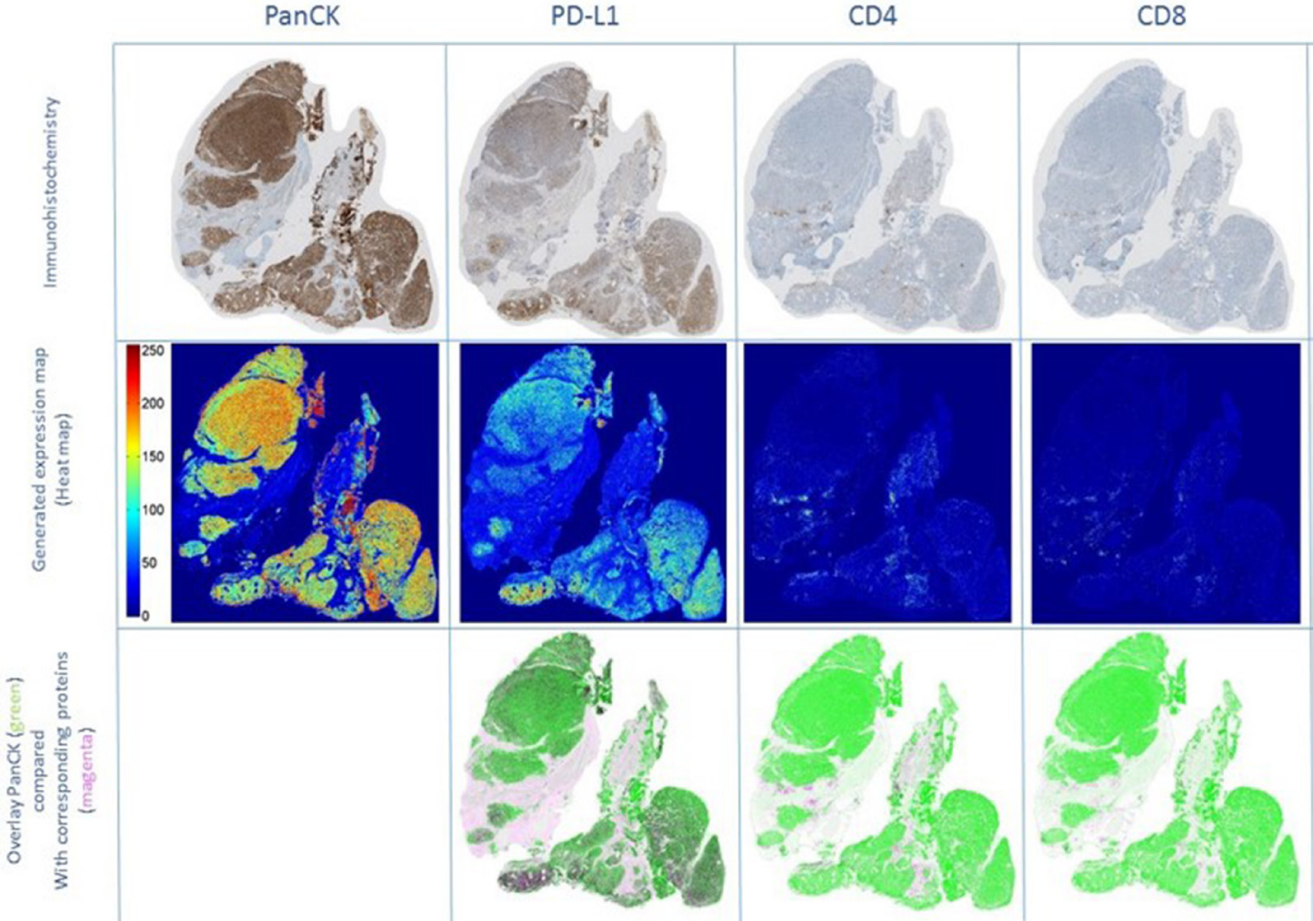
Co-localization experiments using clone E1L3N Immunohistochemical staining for PanCK, PD-L1, CD4, and CD8 on serial sections of a PD-L1 positive CRPC sample (upper row). Immunohistochemical stainings were transformed into expression maps, using false colors for high (red) to low (blue) protein expression (middle row). Overlay images (lower row) were created displaying PanCK as green and PD-L1, CD4, and CD8 proteins as magenta. PD-L1 was co-localized with PanCK positive tumor cell areas but not with the surrounding stroma containing CD4- or CD8-positive lymphocytes. Abbreviations: CD4: cluster of differentiation 4; CD8: cluster of differentiation 8; CRPC: castration resistant prostate cancer; PanCK: pan cytokeratin; PD-L1: programmed cell death ligand 1.

### PD-L1 status and previous treatment modalities

In CRPC samples, expression of PD-L1 (E1L3N) was present in 5 of 81 (6%) cases. All five positive specimens were retrieved by palliative transurethral resection of the prostate (TURP), and no PD-L1 expression was found in bone (*n* = 10), brain (*n* = 1), lung (*n* = 1), or lymph node (*n* = 1) metastases (Supplementary Table 1).

### Co-localization experiments using clone E1L3N

**Figure 6:**
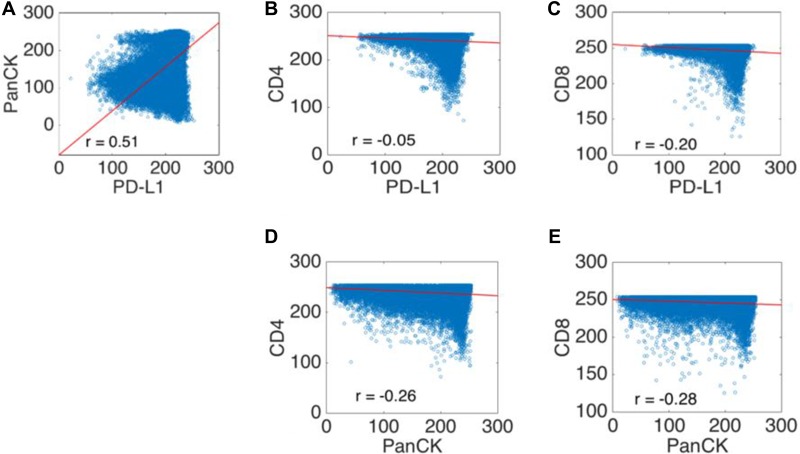
Scatter plots of the co-localization experiments using clone E1L3N PD-L1 and PanCK expression showed a moderate positive Pearson's correlation coefficient indicating overlapping expression areas (**A**). The comparison of PD-L1 and PanCK with CD4 and CD8 immunoreactivity showed negative weak Pearson correlation coefficients, indicating that PD-L1 and PanCK are not expressed in the same location as CD4^+^ or CD8^+^ lymphocytes (**B**–**E**). The x and y axis are represented by (normalized) intensity, ranging from 0 to 255. Abbreviations: CD4: cluster of differentiation 4; CD8: cluster of differentiation 8; PanCK: pan cytokeratin; PD-L1: programmed cell death ligand 1.

Immunohistochemistry revealed co-localization of PD-L1 and PanCK in tumor areas, whereas stroma containing CD4 and CD8 positive lymphocytes was predominantly located surrounding the tumor tissue (Figure 5). This was confirmed by dot-wise correlation showing a moderate positive Pearson correlation coefficient for PD-L1 and PanCK expression (r = 0.51). In addition there were weak negative Pearson correlation coefficients for PanCK and CD4 (r =–0.26) and CD8 (r = −0.28), and for PD-L1 and CD4 (r =–0.05) and CD8 (r = −0.20) expression (Figure 6).

## DISCUSSION

In the present study, we observed no PD-L1 expression in BPH or localized PC, and only limited expression on CRPC TCs and ICs, comparing two different PD-L1 antibodies. We did not observe any association between PD-L1 and previous treatment modalities. We demonstrated how protein expression maps and dot-wise comparison could be a useful objective measurement to describe the relationship between immune cells and tumor-related proteins.

Our findings regarding PD-L1 expression are only partially consistent and extend those from prior reports. PD-L1 expression seems to be an important biomarker as it has been associated with biochemical recurrence in PC [24], and overall survival in other cancers [25]. Furthermore, clinical trials have suggested that tissue expression of PD-L1 might be predictive for therapy response to PD-1 and PD-L1 immunotherapy [12, 14–19, 26–28]. The expression of PD-L1 observed in this study is slightly lower compared to previously published advanced PC [29] and CRPC cohorts (15% and 19% PD-L1 expression respectively) [30], and is distinct to a radical prostatectomy cohort (52.5% PD-L1 expression) [24]. These contradictory results in PD-L1 expression can be explained by different antibodies and scoring methods. Our scoring methods and compared antibodies are in line with recently accepted methodologies and guidelines for non-small cell lung cancer [31] and are currently regarded a common standard for PD-L1 testing.

Several PD-L1 immunohistochemistry assays with custom reagents and scoring systems have been developed in parallel, therefore, studies reporting PD-L1 expression and its value as a biomarker cannot be compared accurately. Whereas the heterogeneous methodologies are addressed by first harmonization studies for lung cancer [32], there is no consensus regarding preferred assay or scoring systems for PC. According to our results, we finally implemented the SP263 antibody for PD-L1 staining at our institution due to its satisfactory staining properties. To improve the described interobserver variability of the different PD-L1 scoring methods [32], digital pathology and protein maps using dot-wise comparison might be a useful tool for pathologists.

The scarce expression of PD-L1 in our CPRC cohort is consistent with the results of several clinical trials, which showed no PD-L1 expression in specimens retrieved before starting anti-PD-L1 treatment [14, 18, 33–35]. However, recently a small cohort of ten CRPC patients treated with pembrolizumab has been published, whereby three patients showed response to pembrolizumab [22]. In two of these three patients, tumor tissue was available and showed PD-L1 expression using the same antibody as in the present study (E1L3N). The authors described that both samples showed a pronounced leukocyte infiltration. Of note is the fact that the lymph node metastasis showed PD-L1 expression in cancer cells (cytokeratin positive), whereas in the liver metastases, PD-L1 expression was found in the infiltrating leukocytes. This early phase II trial [22], along with our results, stress the potential influence of the tumor microenvironment regarding treatment response. Ongoing clinical studies with immunotherapeutic agents in men with CRPC [36] are expected to report PD-L1 expression in pre-therapy specimens, and will hopefully allow for the establishment of harmonized methodologies to assess relevant features.

As PD-L1 expression in other tumor types shows variability at various time points [37], a major question remains if there is a therapeutic window for the optimal timing of immunotherapy, and if PD-L1 expression and response to immunotherapy is associated with previous treatment modalities (e.g. androgen deprivation, radio-, or chemotherapy). The observation in our study that no case after radiotherapy expressed PD-L1 is consistent with a study by Bernstein et al., who showed that treating cell lines with radiotherapy resulted in a decrease in the expression of PD-L1 [38]. Even though radiation therapy seems to decrease PD-L1 expression in this study, there might still be a role for radiotherapy. Further studies with optimal fractionation schemes may be able to induce an immune-stimulatory activity. A thorough understanding of the molecular mechanisms of immune checkpoint regulation will help to make use of this biological knowledge, and to design therapies or combinations with established agents.

These data must be interpreted in the context of the study design. First, although we have analyzed a large cohort, this is a retrospective analysis and there is a potential selection bias. Second, it is difficult to compare the results from our study with previous work due to different methodologies used to evaluate PD-L1 expression. Nevertheless, the positive staining in the included cell lines, our previous publication in germ cell tumors [39], and the accordance of our two antibodies, which was also observed in a harmonization study [32], support our protocol. Third, even though we tried to minimize tumor heterogeneity using two different cores for each patient in the tissue micro arrays (TMA), the real PD-L1 expression might be higher because of heterogeneity of PD-L1 expression within the tumor. Fourth, the therapeutic landscape has changed dramatically in the past years and our cohort was treated with only a subset of the current therapeutic options.

## MATERIALS AND METHODS

### Patients and tissue samples

Clinico-pathological data and follow up information on all patients was retrieved from the Department of Pathology and Molecular Pathology, University Hospital Zurich, and the clinical file of the University Hospital Zurich, Switzerland from 2000 to 2013. The study was approved by the local ethical committee (reference number KEK StV. 25-2008). All patients were treated in different hospitals with the available options: surgical ADT (orchiectomy), chemical ADT (LHRH agonists e.g. goserelin, leuprorelin, anti-androgens (bicalutamide)), chemotherapy (docetaxel) or radiotherapy. Inclusion criteria for this retrospective analysis required tissue collection during transurethral resection of the prostate (TURP), or tumor metastasis resection, and rising PSA or clinical progress under androgen deprivation therapy. The first TMA included 78 formalin-fixed paraffin embedded tissue specimens of patients, which were sampled after the diagnosis of CPRC. Suitable areas for prostate tissue retrieval were marked on routine H&E-stained sections. A section of the paraffin block, 0.6 mm in diameter, was punched out of the suitable area and inserted into the recipient block using a tissue arrayer (Beecher Instruments, Inc., Sun Prairie, WI, USA). The whole tissue microarray preparation was accomplished on the paraffin blocks using cores from two different areas of each tumor or control tissue to account for tissue heterogeneity.

The second TMA consisted of BPH samples (*n* = 70), localized PC (*n* = 96), the androgen-resistant human PC cell lines (PC3 and DU145) and the androgen-sensitive PC cell line (LnCaP). The detailed characteristics of this TMA have been reported previously [40].

### Immunohistochemistry

Three-micron-thick sections of TMA blocks and formalin-fixed, paraffin-embedded tissues were mounted on glass slides (Super-Frost Plus; Menzel, Braunschweig, Germany), deparaffinized, rehydrated and stained with hematoxylin–eosin using standard histological techniques. For immunohistochemical staining of both TMA and large sections, the Ventana Benchmark automated staining system and Ventana reagents (both Ventana Medical Systems, Tucson, AZ, USA) were used. After deparaffinization in xylene, slides were rehydrated in decreasing concentrations of ethanol. Endogenous peroxidase was blocked using the Ventana endogenous peroxidase blocking kit after a rinse with distilled water. For antigen retrieval, slides were heated with cell conditioning solution (CC1, Ventana) according to the manufacturer's instructions.

For the detection of the PD-L1 protein in BPH, PC, and cell line specimens, one anti-human PD-L1 rabbit monoclonal antibody was used: E1L3N (Cell Signaling Technology, Inc., Danvers, MA, USA). For the detection of the PD-L1 protein in CRPC, two anti-human PD-L1 rabbit monoclonal antibodies were used: E1L3N (Cell Signaling Technology, Inc., Danvers, MA, USA), SP263 (Ventana Medical Systems, Tucson, AZ, USA). A multi-tumor TMA and a tonsillectomy specimen were used as positive and negative controls to establish the staining protocol for the PD-L1 antibodies. A dilution of 1:1000 resulted in a strong membranous signal without non-specific background staining in positive controls (squamous epithelium of tonsillectomy specimen). PD-L1 negative lung cancer cases were used as negative controls. For the expression maps, primary antibodies against CD4 (clone SP35 pre-diluted, Ventana Medical Systems, Tucson, AZ, USA), CD8 (clone C8/114B, 1:100, Dako, CA, USA), and PanCK (clone AE1/AE3, 1:50, Dako) were applied and adjusted to the Ventana Benchmark system after performing titrations. Immunohistochemistry with antibodies against Phospho-ERK1/2, Phospho-mTOR, Phospho-4E-BP1, and Ki-67 (MIB1) was performed, as previously described [41].

An experienced pathologist (PJW) evaluated all scanned and digitized tissue microarray spots (Spot browser, Alphelys, Plaisir, France). A second independent observer (CDF) re-evaluated the TMAs. In discrepant cases, the two observers achieved consensus after detailed case discussion. Percentages of PD-L1 positive tumor cells and the staining pattern were evaluated for each punch. PD-L1 expression was recorded if a distinct membranous staining signal on the tumor cell surface was observed. In analogy to the scoring guidelines for non-small cell lung cancer [31], we used a > 1% cutoff for membranous PD-L1 positivity in tumor cells.

### Generation of expression maps

The staining images for CD4, CD8, PanCK, and PD-L1 were aligned, superimposed, and normalized for the generation of a pseudo-colored expression map. Whole slide images of one case, and of all performed stainings were digitized (digital slide scanner NanoZoomer 2.0-HT C9600, Hamamatsu Photonics, Solothurn, Switzerland) at 40x magnification (approximately 2k-3k x 2k-3k pixels per image). The images of consecutive tissue slices were pre-processed and aligned for dot-wise correspondence in four steps. First, the original images were white-balanced to remove grayish background. Second, the tissue regions were cropped and rotated, translated and rescaled for rigid alignment of the consecutive slices to the middle slice. The background was then removed to exclude background artifacts that could disturb the following finer alignment process. Third, to refine the alignment and to address for morphological changes of the tissues in consecutive slices, we incorporated a non-linear SIFT flow algorithm [42]. For SIFT flow, the images were scaled down to half edge size, and the standard parameters were used except for a cell size of 5 pixels and a top window size of 20 pixels to obtain optimal alignment results. Finally, the fully dot-wise aligned and downscaled images were then gray-scaled, inverted, and independently normalized for intensities between 0 and 255. The intensity maps were then compared for each case using a dot-wise Pearson correlation between the pixels of the images. All processes have been implemented in MATLAB R2014a. For visualization purposes only, the gray-scaled images have been pseudo-colored with MATLAB's jet color map function, assigning blue values for low intensities and red values for high intensities.

## CONCLUSIONS

In summary, the scarce PD-L1 expression in CRPC and absence in localized PC indicates that advanced PC patients may have to be selected carefully when offered checkpoint inhibition. However, prospective clinical trial data is needed. Our results do not confirm that PD-L1 expression can be induced by orchiectomy, LHRH-agonists, anti-androgens, radiation therapy or chemotherapy. Neither did we find an association with PD-L1 expression and ERK- or mTOR signaling activity. Protein expression maps and dot-wise comparison could be a useful objective method to describe the topographical relationship between immunological and tumor-related proteins, in general.

## SUPPLEMENTARY MATERIALS TABLE




