# Supplementary material for: Comprehensive immunohistochemical analysis of PD-L1 shows scarce expression in castration-resistant prostate cancer
Source: Oncotarget. 2017 Dec 4;9(12):10284–93. doi: 10.18632/oncotarget.22888 (PMC5828186; doi:10.18632/oncotarget.22888)
Supplement: Supplementary file 1 [file oncotarget-09-10284-s001.pdf]

Comprehensive immunohistochemical analysis of PD-L1 shows scarce expression in castration-resistant prostate cancer

SUPPLEMENTARY MATERIALS

Supplementary Table 1: Clinical data and PD-L1 status. See_Supplementry_Table 1.
