# Supplementary material for: Comprehensive immunohistochemical analysis of PD-L1 shows scarce expression in castration-resistant prostate cancer
Source: Oncotarget. 2017 Dec 4;9(12):10284–93. doi: 10.18632/oncotarget.22888 (PMC5828186; doi:10.18632/oncotarget.22888)
Supplement: Supplementary file 2 [file oncotarget-09-10284-s002.doc]

**Supplementary Table 1: Clinical data and PD-L1 status.**

|  | Age at intervention | Orchiectomy | LHRH agonists | Anti-androgens | Chemotherapy | Radiation of resected tissue | PD-L1 Status (E1L3N) |
| --- | --- | --- | --- | --- | --- | --- | --- |
| Palliative TURP |  |  | 0 |  |  |  |  |
| 1 | 85 |  |  |  |  |  | - |
| 2 | 85 |  |  |  |  |  | - |
| 3 | 85 |  |  |  |  |  | - |
| 4 | 85 |  |  |  |  |  | + |
| 5 | 84 |  |  |  |  |  | - |
| 6 | 84 |  |  |  |  |  | - |
| 7 | 83 |  |  |  |  |  | - |
| 8 | 81 |  |  |  |  |  | - |
| 9 | 80 |  |  |  |  |  | - |
| 10 | 80 |  |  |  |  |  | - |
| 11 | 76 |  |  |  |  |  | - |
| 12 | 75 |  |  |  |  |  | - |
| 13 | 69 |  |  |  |  |  | - |
| 14 | 86 |  |  |  |  |  | - |
| 15 | 82 |  |  |  |  |  | - |
| 16 | 80 |  |  |  |  |  | - |
| 17 | 79 |  |  |  |  |  | - |
| 18 | 67 |  |  |  |  |  | - |
| 19 | 74 |  |  |  |  |  | + |
| 20 | 77 |  |  |  |  |  | - |
| 21 | 75 |  |  |  |  |  | - |
| 22 | 81 |  |  |  |  |  | - |
| 23 | 77 |  |  |  |  |  | - |
| 24 | 77 |  |  |  |  |  | - |
| 25 | 71 |  |  |  |  |  | - |
| 26 | 71 |  |  |  |  |  | - |
| 27 | 71 |  |  |  |  |  | - |
| 28 | 70 |  |  |  |  |  | - |
| 29 | 69 |  |  |  |  |  | - |
| 30 | 68 |  |  |  |  |  | - |
| 31 | 57 |  |  |  |  |  | - |
| 32 | 84 |  |  |  |  |  | - |
| 33 | 81 |  |  |  |  |  | - |
| 34 | 58 |  |  |  |  |  | - |
| 35 | 62 |  |  |  |  |  | - |
| 36 | 71 |  |  |  |  |  | - |
| 37 | 85 |  |  |  |  |  | - |
| 38 | 85 |  |  |  |  |  | - |
| 39 | 80 |  |  |  |  |  | - |
| 40 | 78 |  |  |  |  |  | - |
| 41 | 77 |  |  |  |  |  | - |
| 42 | 73 |  |  |  |  |  | - |
| 43 | 65 |  |  |  |  |  | 2 |
| 44 | 76 |  |  |  |  |  | + |
| 45 | 76 |  |  |  |  |  | - |
| 46 | 74 |  |  |  |  |  | - |
| 47 | 78 |  |  |  |  |  | - |
| 48 | 76 |  |  |  |  |  | - |
| 49 | 74 |  |  |  |  |  | - |
| 50 | 77 |  |  |  |  |  | - |
| 51 | 77 |  |  |  |  |  | - |
| 52 | 76 |  |  |  |  |  | - |
| 53 | 69 |  |  |  |  |  | - |
| 54 | 69 |  |  |  |  |  | - |
| 55 | 80 |  |  |  |  |  | - |
| 56 | 74 |  |  |  |  |  | - |
| 57 | 67 |  |  |  |  |  | + |
| 58 | 68 |  |  |  |  |  | - |
| 59 | 73 |  |  |  |  |  | - |
| 60 | 56 |  |  |  |  |  | - |
| 61 | 54 |  |  |  |  |  | - |
| 62 | 85 |  |  |  |  |  | - |
| 63 | 64 |  |  |  |  |  | - |
| Distant metastasis |  |  |  |  |  |  |  |
| Bone metastasis |  |  |  |  |  |  |  |
| 1 | 80 |  |  |  |  |  | - |
| 2 | 66 |  |  |  |  |  | - |
| 3 | 63 |  |  |  |  |  | - |
| 4 | 61 |  |  |  |  |  | - |
| 5 | 63 |  |  |  |  |  | - |
| 6 | 67 |  |  |  |  |  | - |
| 7 | 76 |  |  |  |  |  | - |
| 8 | 81 |  |  |  |  |  | - |
| 9 | 61 |  |  |  |  |  | - |
| 10 | 68 |  |  |  |  |  | - |
| Brain metastasis |  |  |  |  |  |  |  |
| 1 | 64 |  |  |  |  |  | - |
| Lung metastasis |  |  |  |  |  |  |  |
| 1 | 75 |  |  |  |  |  | - |
| Visceral metastasis |  |  |  |  |  |  |  |
| 1 | 62 |  |  |  |  |  | - |
| Lymph node |  |  |  |  |  |  |  |
| 1 | 68 |  |  |  |  |  | - |
|  |  |  |  |  |  |  |  |

All tissues of castration-resistant prostate cancer (CRPC) enlisted according to tissue origin marked with a black box if responding therapy was received. Light grey indicating weakly positive and dark grey strong immunohistochemistry staining.

TURP Transurethral resection of the prostate; PD-L1 **Programmed Death Receptor Ligand; LHRH** Luteinizing hormone-releasing hormone
